# P-731. Viruses Associated with Acute Respiratory Illness in a Community Cohort of Children and Adults, Oregon and Washington, United States, 2022 - 2023

**DOI:** 10.1093/ofid/ofae631.927

**Published:** 2025-01-29

**Authors:** Glen Abedi, Jade James-Gist, Tara M Babu, Melissa Briggs-Hagen, Cassandra Boisvert, Marco Carone, Janet A Englund, Helen Y Chu, Leora R Feldstein, Peter D Han, Jennifer L Kuntz, Natalie K Lo, Richard A Mularski, Allison L Naleway, Ian D Plumb, Mark A Schmidt, Ning Smith, Lea Starita, Ana A Weil, Neil D Yetz, Claire Midgley

**Affiliations:** Centers for Disease Control and Prevention, Atlanta, Georgia; CDC, Atlanta, Georgia; University of Washington, Seattle, Washington; Centers for Disease Control and Prevention, Atlanta, Georgia; Kaiser Permanente, Portland, Oregon; University of Washington, Seattle, Washington; Seattle Children’s Hospital, Seattle, Washington; University of Washington, Seattle, Washington; Centers for Disease Control and Prevention, Atlanta, Georgia; University of Washington, Seattle, Washington; Kaiser Permanente Center for Health Research, Portland, Oregon; University of Washington, Seattle, Washington; 1. Kaiser Permanente Center for Health Research, Portland, Oregon, Portland, Oregon; Kaiser Permanente Center for Health Research, Portland, Oregon; Division of Foodborne, Waterborne, and Environmental Diseases, Centers for Disease Control and Prevention, Atlanta, GA, Atlanta, Georgia; Center for Health Research, Kaiser Permanente Northwest, Portland, Oregon; Kaiser Permanente Center for Health Research, Portland, Oregon; Brotman Baty Institute for Precision Medicine; University of Washington, Seattle, Washington; University of Washington, Seattle, Washington; Kaiser Permanente Center for Health Research, Portland, Oregon; Centers for Disease Control and Prevention, Atlanta, Georgia

## Abstract

**Background:**

Testing for most respiratory viruses in the U.S. occurs in limited settings, resulting in an incomplete understanding of the full burden of disease, especially among non-medically-attended infections. We used a U.S. community-based cohort of participants aged 6 months – 49 years in Washington and Oregon to assess respiratory virus detections and outcomes in participants with acute respiratory illness (ARI).

**Table 1.**
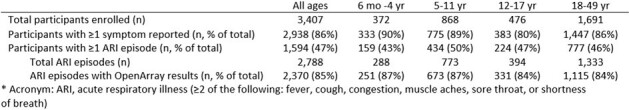
Number of participants enrolled in CASCADIA and acute respiratory illness* episodes, by age group.

**Methods:**

At symptom onset, enrolled participants completed symptom surveys and self-collected nasal swabs. At days 7 and 14 post symptom onset, they completed follow-up symptom surveys. Swabs were tested for multiple viruses using TaqMan RT-PCR (OpenArray, ThermoFisher). We assessed viral detections and outcomes among ARI episodes during September 2022 — August 2023. ARI was defined as ≥ 2 of the following: fever, cough, congestion, muscle aches, sore throat, or shortness of breath. A distinct ARI episode was defined as a reported onset of a new illness > 30 days after a prior onset for the same participant. For each episode, we included test results from all swabs collected 0 – 7 days post-onset.

**Figure 1.**
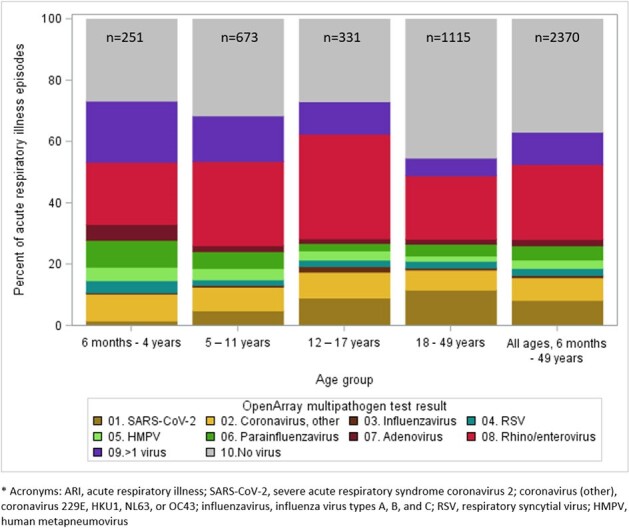
Proportion of viruses detected among acute respiratory illness episodes with multi-pathogen OpenArray test results* (n=2,370), by age group.

**Results:**

Among 3,407 participants enrolled during the analytic period (median age 17 years, IQR: 9 – 41; 59% female sex; 70% non-Hispanic White), we identified 2,788 ARI episodes, of which 2,370 (85%) had OpenArray virus test results (**Table 1**). Of these 2,370 episodes, 1,248 (53%) had a single virus detected, 249 (11%) had > 1 virus (i.e. viral codetections), and 873 (37%) had none; these results varied by age (**Fig.1**), and detected virus varied by month (**Fig. 2**). Among 1,248 detections of single viruses, rhinovirus/enterovirus (46%) and SARS-CoV-2 (16%) were detected most frequently; SARS-CoV-2 was more common in adults than children. Participant characteristics, symptoms, and illness outcomes are presented by virus type in **Table 2**. Healthcare was sought for 13% (range: 6 – 19% across viruses) of ARI episodes and missed work or school was reported for 26% (range: 20 – 45% across viruses).

**Figure 2.**
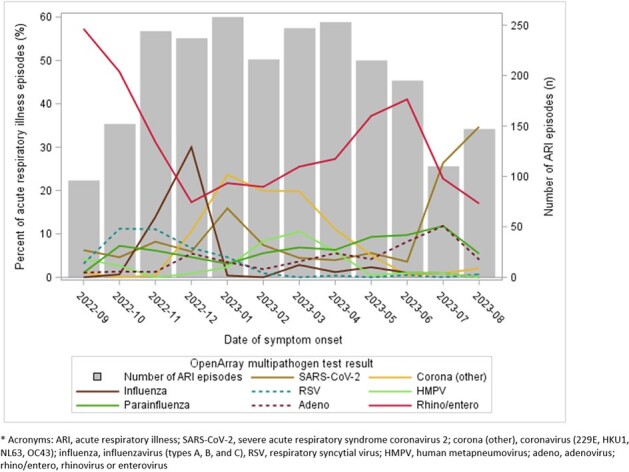
Number of acute respiratory illness episodes (n=2,370) and proportion positive on multi-pathogen OpenArray test*, by detected virus and month. Virus percentages include single detections and codetections.

**Conclusion:**

Virus-associated ARI varied by age, and outcomes varied by virus, but healthcare needs and missed work/school were reported for all viruses. These data may inform clinical and socioeconomic burden estimates for respiratory viruses.

**Table 2.**
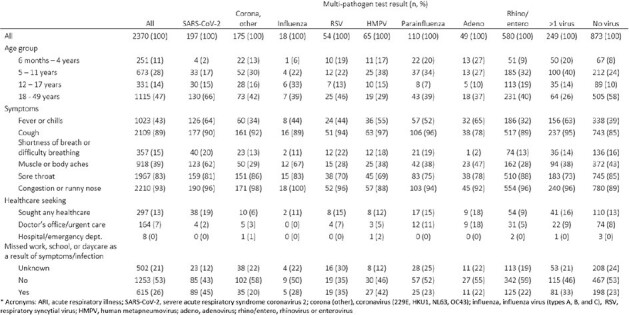
Age groups, clinical, and outcome characteristics of acute respiratory illness episodes (n=2,370), by detected virus*.

**Disclosures:**

**Jade James-Gist, MPH**, NA: na **Janet A. Englund, MD**, Abbvie: Advisor/Consultant|AstraZeneca: Advisor/Consultant|AstraZeneca: Grant/Research Support|GlaxoSmithKline: Advisor/Consultant|GlaxoSmithKline: Grant/Research Support|Meissa Vaccines: Advisor/Consultant|Merck: Advisor/Consultant|Pfizer: Board Member|Pfizer: Grant/Research Support|Pfizer: Speaker at meeting|SanofiPasteur: Advisor/Consultant|Shinogi: Advisor/Consultant **Helen Y. Chu, MD, MPH**, Abbvie: Advisor/Consultant|Merck: Advisor/Consultant|Vir: Advisor/Consultant **Jennifer L. Kuntz, MS, PhD**, Hillevax, Inc: Grant/Research Support **Richard A. Mularski, MD, MSHS, MCR**, Pfizer, Inc: Grant/Research Support **Mark A. Schmidt, PhD, MPH**, HilleVax: Grant/Research Support|Janssen: Grant/Research Support|Moderna: Grant/Research Support|Pfizer: Grant/Research Support|Vir Biotechnology: Grant/Research Support **Neil D. Yetz, M.P.H.**, N/A: Expert Testimony

